# Expansion of Tick-Borne Rickettsioses in the World

**DOI:** 10.3390/microorganisms8121906

**Published:** 2020-11-30

**Authors:** Mariusz Piotrowski, Anna Rymaszewska

**Affiliations:** Institute of Biology, University of Szczecin, 70-453 Szczecin, Poland; anna.rymaszewska@usz.edu.pl

**Keywords:** Tick-borne rickettsioses, Tick-borne diseases, Rickettsiales

## Abstract

Tick-borne rickettsioses are caused by obligate intracellular bacteria belonging to the spotted fever group of the genus *Rickettsia*. These infections are among the oldest known diseases transmitted by vectors. In the last three decades there has been a rapid increase in the recognition of this disease complex. This unusual expansion of information was mainly caused by the development of molecular diagnostic techniques that have facilitated the identification of new and previously recognized rickettsiae. A lot of currently known bacteria of the genus *Rickettsia* have been considered nonpathogenic for years, and moreover, many new species have been identified with unknown pathogenicity. The genus *Rickettsia* is distributed all over the world. Many *Rickettsia* species are present on several continents. The geographical distribution of rickettsiae is related to their vectors. New cases of rickettsioses and new locations, where the presence of these bacteria is recognized, are still being identified. The variety and rapid evolution of the distribution and density of ticks and diseases which they transmit shows us the scale of the problem. This review article presents a comparison of the current understanding of the geographic distribution of pathogenic *Rickettsia* species to that of the beginning of the century.

## 1. Introduction

Tick-borne rickettsioses are caused by obligate intracellular Gram-negative bacteria belonging to the spotted fever group (SFG) of the genus *Rickettsia*. These infections are among the oldest known diseases transmitted by vectors [1]. In the last three decades there has been a rapid increase in the recognition of this disease complex. This unusual expansion of information was mainly caused by the development of molecular diagnostic techniques that have facilitated the identification of new and previously recognized rickettsiae. A lot of currently known bacteria of the genus *Rickettsia* have been considered nonpathogenic for years, and moreover, many new species have been identified with unknown pathogenicity. Likewise, a lot of rickettsioses, which were attributed to one species of tick or one geographical area, are nowadays identified in many vectors and in different regions of the world. There are several classifications of bacteria of the genus *Rickettsia*, but the most commonly used one divides them into four groups: spotted fever group (SFG), typhus group (TG), the *Rickettsia bellii* group, and the *Rickettsia canadensis* group [1,2].

## 2. Epidemiology

**Figure 1:**
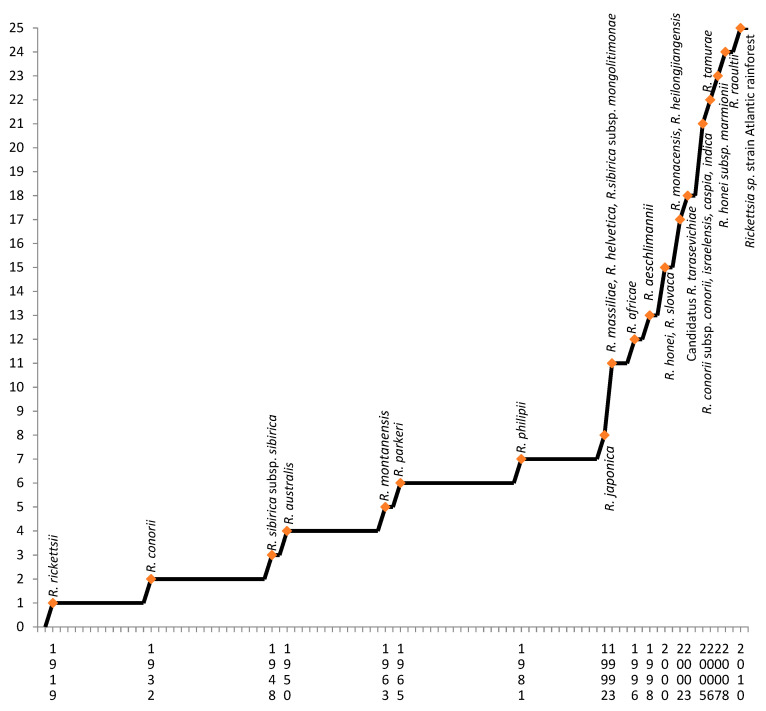
The number of pathogenic species of bacteria belonging to the genus *Rickettsia* known in particular years. The first publication with description of the species was considered as the date.

At present, there are 25 known pathogenic species of bacteria belonging to the genus *Rickettsia*, most of which have been described for the first time in the last 30 years (Figure 1). The main vector of rickettsioses are ticks of the genus *Ixodes* [1]. The newly discovered rickettsiae have changed our understanding of the clinical features and epidemiology of these bacteria. Besides the classic symptomatic triad, i.e., fever, rash, and headache, it has been shown that each rickettsiosis is characterized by specific features, including the severity and inoculation rate. Unlike, for example, Mediterranean spotted fever (MSF), which describes the occurrence of one eschar at the site of a tick bite, patients with African tick bite fever (ATBF) have several eschars. The SENLAT (Scalp Eschar and Neck Lymphadenopathy after Tick) syndrome is characterized by the appearance of an eschar on the scalp with the simultaneous enlargement of the cervical lymph nodes. In the case of Rocky Mountain spotted fever (RMSF), a rash occurs all over the body including skin of the palms and soles [3]. The rash can be maculopapular, as in the case of RMSF, MSF, or many other rickettsioses, vesicular as in many cases of ATBF, purple, or absent. An eschar created at the site of a tick bite is a symptom characteristic for many SFG rickettsioses but may be completely absent as in the case of RMSF. Rickettsioses may be mild, severe, or even fatal [4].

Globalization and climate warming are factors that can affect the increase in transmission of rickettsioses by ticks. Higher temperatures, on the one hand, influence the aggressiveness of ticks and their tendency to attack humans, and on the other hand, are the cause of infection cases at unusual times of the year for these infections [3,5]. In addition, the increasing trend of travelling to more and more exotic parts of the world increases the potential exposure to ticks transmitting diseases including rickettsioses [5,6].

Epidemiology of any rickettsial disease transmitted by ticks reflects the geographical distribution and seasonal activity of tick vectors and their hosts involved in the transmission of these pathogens, as well as human behaviors that expose people to ticks attachment and subsequent infection. The distribution of tick-borne rickettsioses varies geographically and is similar to the original distribution of tick vectors, which makes it important for doctors to be familiar with the regions where tick rickettsiae often occur. Healthcare and veterinary specialists should be aware of changes in the distribution of vectors, and should be informed about newly emerging and newly identified pathogens transmitted by ticks [7].

### 2.1. Emergence of Rickettsioses

Most of the discovered pathogenic species of rickettsiae were first identified in ticks. Years later they were associated with disease symptoms in humans and animals. *Rickettsia parkeri* was first identified in 1937 in *Amblyomma maculatum* ticks at the Gulf of Mexico coast, and the first infection caused by this rickettsia was described after almost 70 years [8]. *Rickettsia slovaca* was first isolated in 1968 from *Dermacentor marginatus* ticks in Czechoslovakia, and the first documented case of human infection was reported several years later [9]. Taking into account the difficulties in predicting the pathogenicity of new species of rickettsiae isolated from arthropods, these species should always be considered potentially pathogenic to humans [3].

Rickettsioses belong to the oldest known diseases transmitted by vectors. However, the range and importance of the recognized tick-related pathogenic rickettsiae have increased dramatically over the past 30 years, which makes this disease complex an ideal paradigm for the understanding of emerging and reemerging infections (Figure 1) [6].

In areas endemic for ticks, common activities such as playing in the yard, visiting the nearest park, walking the dog or working in the backyard garden are potential sources of exposure to ticks. Depending on the tick species, many types of environments serve as their potential habitat. Areas with high uncut grass, with numerous weeds and rich plant litter, may pose a high risk for the presence of ticks, however, they can also be found in well-kept green areas. Moreover, some species can tolerate drier conditions and can be found in areas free of vegetation or covered only with litter or needles. Frequent places where ticks may occur are roadside vegetation, trails, yards or fields, urban and suburban recreational parks, or golf courses. Activities that usually result in contact with potential tick habitats are recreational activities such as camping, hiking, fishing, hunting, gardening, running or walking, and professional activities such as forestry, agriculture, land development, or military exercises [7]. It is assumed that ticks live in forests far from human farms, but research confirms that environmental conditions in cities do not differ from those that they have in wild nature and are equally suitable for them. Ticks are widespread throughout the world and their dynamics is closely related to environmental conditions [10].

### 2.2. Ticks as Vectors and Reservoirs

Ticks are the absolute parasites of vertebrates. Two tick families can transmit a wide range of pathogens. They include *Ixodidae* (hard ticks), now covering more than 700 species worldwide [11], and *Argasidae* (soft ticks), covering approximately 200 species [12]. Ticks are the most important vectors and reservoirs of rickettsiae in almost every region of the world. Vector skills of ticks are increased by the use of many hosts, feeding of various developmental stages (larva, nymph, imago), and a long life (sometimes longer than the host). Some species of rickettsiae are particularly associated with one species of tick, while others occur in different species of these arthropods (Table 1). The risk of rickettsioses in a given region may depend on the presence of specific species of ticks, their number, time of attachment to the host, human activity, and geographical and climatic conditions [3]. Ticks are characterized by low mobility and, in comparison to flying vectors, they invest less energy in finding a host, and can therefore survive for a long period, in some cases even for many years. In comparison with other vectors, they take in significantly more food, which also contributes to the increase of their vector capabilities. In most cases, every developmental stage feeds only once before the moulting, but for a long time, which in extreme cases can reach up to 14 days [5].

It is thought that many species of bacteria of the genus *Rickettsia* are transmitted vertically among ticks, which suggests that ticks are not only vectors but also reservoirs of rickettsiae in nature [13,14]. The vertical transmission of rickettsiae in arthropods helps to maintain infections in nature, but for some *Rickettsia* species, the life cycle of an infected arthropod with the infection of one or several vertebrates is a guarantee of survival of bacteria in the environment [1]. It is believed that humans are only occasional carriers of ticks and do not play a role in the circulation of the bacteria in nature [15].

### 2.3. Distribution of Ticks

Distribution of ticks is highly dependent on their biotopes, and very few species of ticks are dispersed all around the world. Ticks have a relatively low mobility, and their spread is associated mainly with the migration of host animals, especially birds that travel thousands of kilometers, often to other continents [3]. General tolerance to extreme temperatures and tick drying varies depends on the species, developmental stage, sex, age, and physiological state, and it is very difficult to characterize the general survival frames for all ticks. Almost all ticks show seasonal variability of activity, differing between species and developmental stages. Most ticks of the genus *Ixodidae* spend up to 99% of their lives off the host, except for one- and two-host ticks, which normally live in harsh environments where off-host survival is difficult [5].

Two main factors are crucial for the presence of ticks: suitable environmental conditions and the availability of appropriate hosts. There are several trends in the development of European cities affecting the above factors. The tendency to preserve green areas in cities is a positive aspect both for human life and the life of ticks. In many cities there are green areas, such as urban forests, parks, wide boulevards and old cemeteries, as well as large private properties in suburban areas. Focusing on environmental protection, ecological standards of urban development and creation of residential districts bordering with natural areas create favorable conditions for the existence of a population of ticks. With the development and expansion of urban areas, on the one hand, there is an increase in the number of urban populations of domestic animals, and on the other hand, there is an increasing frequency of migration of wild animals to the territorial boundaries of cities. This phenomenon is conducive to the existence of ticks in the direct vicinity of human farms [49].

Habitats of people, animals and ticks overlap. Understanding of the habitats where ticks may be encountered is important for preventing diseases that are transmitted by them. The locations of these vectors can vary a lot depending on the biology of ticks and the biology of host. Ticks are characterized by their different abilities to dry out. *Ixodes* sp. require humid environments, while *Rhipicephalus sanguineus* can survive high temperatures and low humidity. *Dermacentor variabilis* occur in meadows covered with forest, while *Amblyomma americanum* can live in dry forests. *Rhipicephalus sanguineus* ticks are commonly found in homes and their surroundings. Some ticks look for their hosts waiting in grass or on deciduous vegetation, while others are found in forest litter or fallen needles. Some ticks actively look for their hosts, while others wait for their host to pass by. The large diversity of habitats makes their avoidance very difficult [7].

### 2.4. Rickettsial Pathogenicity

*Rickettsia ricketsii* was first identified in 1919, although the disease entity has been known since 1906. *R. rickettsi* causes a disease called Rocky Mountain spotted fever (RMSF). RMSF is manifested by the sudden onset of high fever, which is accompanied by headaches, nausea, vomiting, and myalgia. In rare cases, an inoculation eschar may occur. A characteristic rash appears within two to four days after the onset of fever [50], and is manifested as small pink spots on the wrists, ankles, and forearms and gradually covers other parts of the body. In 50% to 60% of patients, these changes are transformed into ecchymosis or purpura. In severe cases, pulmonary edema and haemorrhage, cerebral edema, myocarditis, renal failure, disseminated intravascular coagulation (DIC), and gangrene may occur. In rare cases, there are jaundice, central nervous system damage, respiratory failure, and acute renal failure. Despite the availability of effective treatment, it is estimated that 5% to 10% of patients in the United States die [51]. These rates are higher in other countries and the figures show a 38% case-fatality rates in Mexico [52], 20% to 40% in Brazil, and in some areas even up to 80%, where diagnosis and antibiotic treatment may be delayed [53]. In Panama, out of six documented cases, 100% mortality was demonstrated [54]).

*Rickettsia conorii* subsp. *conorii* was first identified in 1932, and in the same year the first disease case called Mediterranean spotted fever (MSF) was described. In most MSF cases, six days after infection, the following symptoms suddenly appear: fever (94% to 100% of cases), flu-like symptoms (78% of cases), and extreme exhaustion (64% of cases). Frequently, an eschar appears at the tick-bite site (53% to 77% of cases) and rash spreads on hands and soles of the feet (87% to 96% of cases). The rash in 94% of cases is maculopapular, but in 6% of cases an ecchymotic form was noted [55]. In some cases, multiple eschar may occur, which most often appear in infected children [56]. The factors that may result in a more serious course of the disease include: advanced age, reduced immunity, alcoholism, previous recommendation of inappropriate antibiotics, and delayed treatment [1]. A more serious life-threatening course may be characterized by: cardiac symptoms (coronary artery ectasia, myocarditis, atrial fibrillation) [57,58], ocular symptoms (uveitis, retinopathy, retinal vasculitis) [59,60], neurological symptoms (ischemic stroke, meningitis, hearing loss) [55], and moreover, pancreatitis [61], splenic rupture, acute renal failure, the presence of hemophagocytic syndrome [1], or acute respiratory distress syndrome [62]. Deaths were reported in France, Greece, Bulgaria, and Turkey, and fatal cases in Portugal reached 13% [1]. Death rate on the African continent is estimated at 3.6%, however, in patients hospitalized with neurological and multiorgan symptoms, it reached up to 54.5% [63,64].

*Rickettsia conorii* subsp. *israelensis* causes a disease called Israeli spotted fever (ISF) or Israeli tick typhus, in which first case was described in 1971 in Israel. The clinical symptoms of the disease are very similar to MSF. The occurrence of an inoculation eschar is less frequent (38%), while gastrointestinal problems such as nausea (63% of cases) and vomiting (56% of cases) are more frequently reported. The fatality rate of recorded cases in Portugal was up to 29% [65].

*Rickettsia conorii* subsp. *caspia* causes a disease called Astrakhan fever, which had its first cases recorded in the Astrakhan region and regions near the Caspian Sea. Clinical symptoms are very similar to the course of MSF. An inoculation eschar occurs only in 23% of cases, maculopapular rash is reported in 91% of cases, and in 20%, solitary elements are transformed into petechiae. At the peak of the fever, the occurrence of nose bleeding and bleeding from the injection sites after medication have been recorded [1].

*Rickettsia conorii* subsp. *indica* is a factor that causes a disease called Indian tick typhus. The clinical picture of the disease is similar to the course of MSF, with the difference being that a purple rash is often reported, and the occurrence of an inoculation eschar is rare [66]. Complications in the form of gangrene have been also found [67].

*Rickettsia parkeri* is a bacterium whose first case of infection caused in humans was described in 2004. Infections have also been reported in dogs and cows [1]. Most cases are characterized by necrotic rash occurring a few days after the infected tick-bite. After a few days there is a fever with intensity from low to moderate. An eschar may occur at the bite site. The infections caused by *R. parkeri* have a milder course than RMSF and no fatal cases have been reported so far [1,68].

*Rickettsia massiliae* is a bacterium that was first isolated in 1992 near the city of Marseille in France. Symptoms reported by patients are similar to those during MSF. In addition, a case of visual loss and bilateral chorioretinitis have been reported [1]. The pathogen was also detected in a tourist who returned from Buenos Aires (Argentina), and the symptoms he developed were fever, purple rash on the upper and lower limbs, and an eschar on the right leg [69]. The infections with this bacterium were reported in dogs as well [70].

*Rickettsia africae* causes a disease called African tick bite fever (ATBF). Symptoms appear within five to seven after a tick bite with sudden fever, headache, fatigue, and myalgia. An eschar appearing at the site of a tick bite is reported in 50% to 100% of cases, but quite often there are multiple eschars. Other common symptoms of the disease are lymphadenopathy, maculopapular or vesicular rashes, appearing in 88% of cases and sporadic aphthous stomatitis. Generally, the disease is not severe, although there have been reports of cardiomyopathy, neuropathy, cellulitis, and chronic fatigue [1]. Furthermore, ATBF is the second disease after malaria diagnosed among tourists returning from a trip to sub-Saharan Africa [71,72].

*Rickettsia philipii* is a bacterium whose infection was first described in 2008 in a patient in North Carolina in the USA, although the bacterium has been known since 1975 when it was isolated from *Dermacentor occidentalis* in South Carolina (Rickettsia 364D). It is believed that many cases may have been misdiagnosed as RMSF, and the characteristic symptoms of this infection include the occurrence of an eschar, fever, headache, myalgia, and general fatigue [73].

*Rickettsia montanensis* was first isolated in 1961 from ticks collected in eastern Montana, USA. There were no confirmed cases of infection in humans, however, there are data published in 2012 informing that a child who was symptomatic with rash in the past was bitten with a tick infected with *R. montanensis*. This case indicates that this bacterium may cause a mild illness similar to spotted-fever-like diseases [74].

*Rickettsia* sp. strain Atlantic rainforest (or strain Bahia) is a bacterium responsible for two cases with clinical symptoms similar to those caused by *R. parkeri*, which manifested with a sub-febrile state, enlarged lymph nodes, and the occurrence of an inoculation eschar [75,76].

*Rickettsia sibirica* subsp. *mongolitimonae* was first isolated from ticks collected on the territory of Mongolia. The disease entity is called LAR (lymphangitis-associated rickettsiosis). Typical symptoms include fever (100% of cases), headache (86% of cases), myalgia (90% of cases), rash (77% of cases), enlargement of lymph nodes (71% of cases), lymphangitis (43% of cases) and a single or multiple inoculation eschars (92% of cases). There were no fatal cases, but complications such as acute renal failure, retinitis, and lethargy with hyponatraemia were observed [77,78].

*Rickettsia slovaca* is a bacterium that causes a disease called TIBOLA (Tick-Borne Lymphadenitis*)* or DEBONEL (Dermacentor-Borne Necrosis Erythema Lymphadenopathy). After investigations from 2010 and 2011, when similar symptoms were confirmed related to the infections with *Bartonella henselae* and *Francisella tularensis*, a unified name was proposed for this disease entity; SENLAT (Scalp Eschar and Neck Lymphadenopathy After Tick) [79,80]. The incubation period of the disease is one to 15 days. The clinical picture of the infection caused by *R. slovaca* includes asthenia (70% of cases), headaches (53% of cases), painful enlargement of the cervical lymph nodes (69%–100% of cases), painful inoculation eschar on the scalp (64% of cases), increased body temperature (36%–54% of cases), rash (5% of cases), and in a few cases, face edema. Treatment goes well, but very often there is an alopecia around the eschar for a few months (59% of cases) and long-term asthenia (14% of cases) [81,82].

*Rickettsia raoultii* is a bacterium which, similar to *R. slovaca*, is associated with the development of the disease called TIBOLA or DEBONEL. Similar to as *R. slovaca*, it is responsible for causing the disease called SENLAT [79,80]. The disease symptoms are very similar to those caused by *R. slovaca,* with the difference that there are no cases of alopecia [82,83].

*Rickettsia monacensis* is a bacterium whose first cases of human infection were found in 2005 in Spain and Italy. In addition to flu-like symptoms and fever, some of the patients found an inoculation eschar, as well as a rash also covering the hands and soles of the feet. After treatment, patients recovered [84,85].

*Rickettsia aeschlimannii* is a bacterium whose first case of infection was found in France in a patient returning from Morocco. Clinical symptoms are similar to MSF [86].

*Rickettsia helvetica* is a bacterium that causes an infection with a relatively mild course, with headache, sometimes rash and an inoculation eschar [86].

*Rickettsia sibirica* subsp. *sibirica* is a bacterium causing the disease called Siberian tick typhus (STT) and it was described for the first time in 1930 in Russia. The incubation period of the disease is four days, and the symptoms are characteristic for spotted fevers, i.e., high fever, inoculation eschar, lymphadenopathy and maculopapular (rarely ecchymotic) rash. The disease usually has a mild course and is rarely associated with serious complications [1].

*Rickettsia heilongjiangensis* is a bacterium that causes a disease called Far- eastern spotted fever (FESF). The first cases were recorded in the Russian Far East and the People’s Republic of China where they were initially misdiagnosed as the STT [1]. Typical symptoms are fever, headache and dizziness, shivering, myalgia, weight loss and spotted or maculopapular rash, enlargement of the lymph nodes, and an inoculation eschar [18].

*Rickettsia japonica* is an etiological factor of Japanese spotted fever (JSF), whose characteristic symptoms are fever, headache, rash, and the occurrence of an eschar at the tick bite site [87].

*Rickettsia honei* is a bacterium causing the disease called Flinders Island spotted fever (FISF) described for the first time in 1991. Rickettsiosis caused by this bacterium generally has a mild course with symptoms such as fever, headache and myalgia, coughing and a maculopapular rash (with no signs of vesicularity). In the course of the infection, encephalitis, pneumonia, tinnitus, and deafness have been also reported [1].

*Rickettsia tamurae* is a bacterium that was first isolated in 1993 from *Amblyomma testudarium* ticks in Japan [88], but officially as a species it was recognized in 2006 [89]. The first case of infection was recorded in Japan in 2011. A symptom described in the course of infection was local skin inflammation with edema. Although no other typical symptoms for rickettsiosis were found, it should be assumed that this bacterium has pathogenic potential [90].

*Rickettsia australis* is a bacterium responsible for causing the disease called Queensland tick typhus (QTT), whose first cases were reported in 1946 [1]. The QTT symptoms are fever, headache, malaise, shivering, maculopapular rash, and inoculation eschar. The course of the disease varies from mild to life-threatening [91].

*Rickettsia honei* strain *marmionii* is a bacterium that was first described in 2005 in Australia [92], and the disease entity caused by it is called the Australian spotted fever [1]. The clinical picture is similar to that in the FISF and is characterized by fever, headache, myalgia, coughing, maculopapular rash, nausea, sore throat, enlarged lymph nodes, and an eschar appearing at the tick bite site [93].

Candidatus *Rickettsia tarasevichiae* is a bacterium whose first illness case was recorded in 2013. Symptoms of the infection include fever, asthenia, weight loss, nausea, headache, enlarged lymph nodes and the occurrence of an inoculation eschar. However, no rash was observed. There was also a fatal case in a patient after coma, renal function disorders and respiratory acidosis [94].

The above-mentioned bacteria of the genus *Rickettsia* are those whose disease-related properties have been confirmed. However, there are many species or “candidates” for new species, which are considered to be potentially pathogenic, although the literature data do not allow definitely putting forward such a thesis. These include: *R. rhipicephali*, Candidatus *R. barbariae*, Candidatus *R. amblyommii*, Candidatus *R. andeanae*, Candidatus *R. colombianensi*, Candidatus *R. cooleyi*, Candidatus *R. kellyi*, Candidatus *R. kotlanii*, Candidatus *R. kulagini*, Candidatus *R. liberiensis*, Candidatus *R. moreli*, Candidatus *R. principis*, Candidatus *R. rioja*, Candidatus *R. siciliensis*, Candidatus *R. vini*, *R. antechini*, *R. argasii*, *R. asiatica*, *R. derrickii*, *R. gravesii*, *R. guntherii*, *R. hoogstraalii*, *R. peacockii, R. sauri*, *R.* sp. clone KVH-02-3H7, *R.* sp. COOPERI, *R.* sp. NOD, *R.* sp. strain Davousti, *R.* sp. strain DmS1, *R.* sp. strain IXLI1, *R.* sp. strain Uilenbergi, *R. tasmanensis*, *R. monteiroi* [1], and Candidatus *R. wissemanii* [95].

The multiplicity of species belonging to the genus *Rickettsia* may be surprising, but it should be noted that the bacterial taxonomy, especially *Rickettsia* spp., is still under discussion. It is accepted that a new species determines at least 1.3% difference in the nucleotide sequence for 16S rRNA [96]. However, this rule does not work in the case of rickettsiae belonging to the SFG group because their similarity is 97.9% to 99.8%. Therefore, the sequences of genes such as *gltA*, *ompA*, *ompB* or D gene are additionally used for the description of new species. Nevertheless, although the sequences show a larger diversity between certain species, the similarity is still more than 99% [97].

### 2.5. Geographical Distribution of Rickettsia spp.

Ticks, just after mosquitoes, being the second vectors of transmitted diseases are responsible for the transmission of viruses, bacteria, fungi, and protozoa. From a medical and veterinary point of view, most of the approximately 100 arthropod-transmitted infections can be associated with 116 species of ticks (32 species of *Argasidae* and 84 species of *Ixodidae*). The global distribution of ticks and rickettsioses can introduce new species of rickettsiae to new geographical areas [10]. Molecular tests have shown that bacteria of *Rickettsia* genus are distributed all over the world. Many *Rickettsia* species are present on several continents. The geographical distribution of rickettsiae is related to their vectors. In addition, the occurrence of rickettsiae may be influenced by interactions between *Rickettsia* species in the same tick species or in an individual tick. Non-pathogenic or slightly pathogenic *Rickettsia* may have a negative effect on virulent species by competing for limited microhabitats in a host [3].

**Figure 2:**
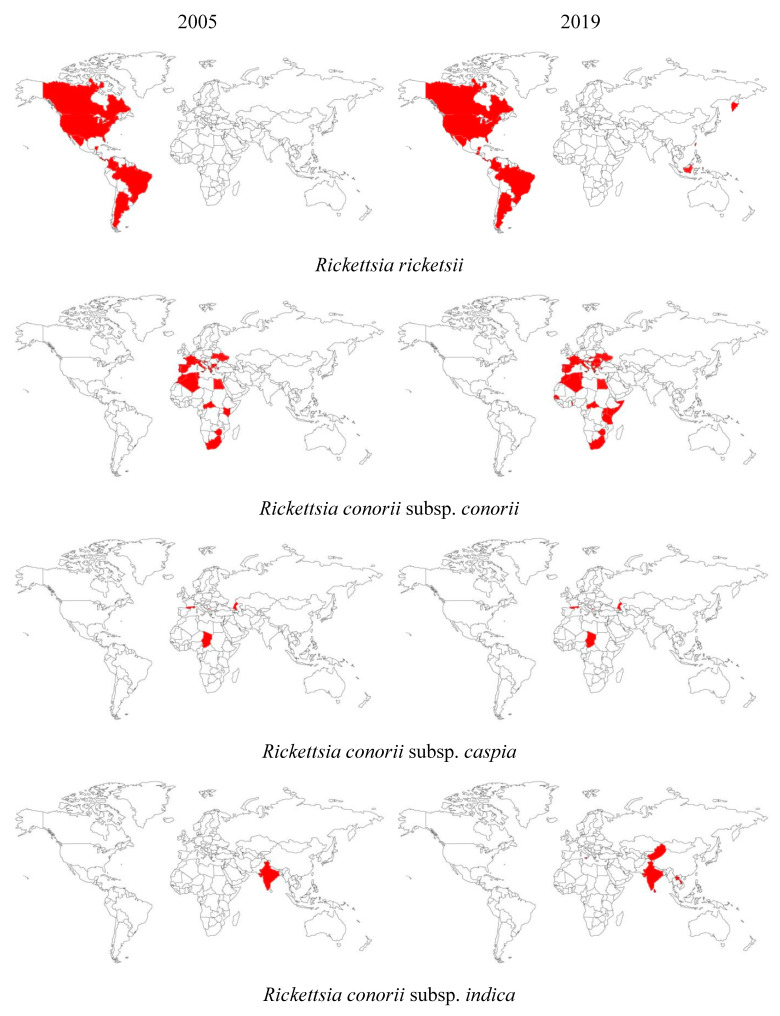

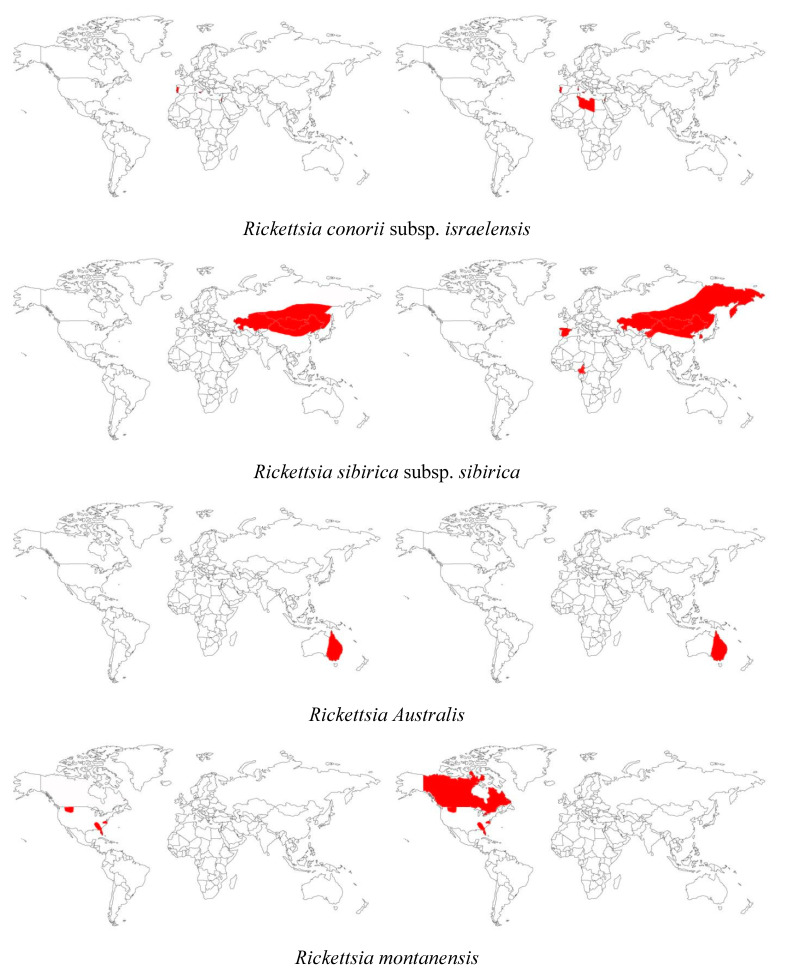

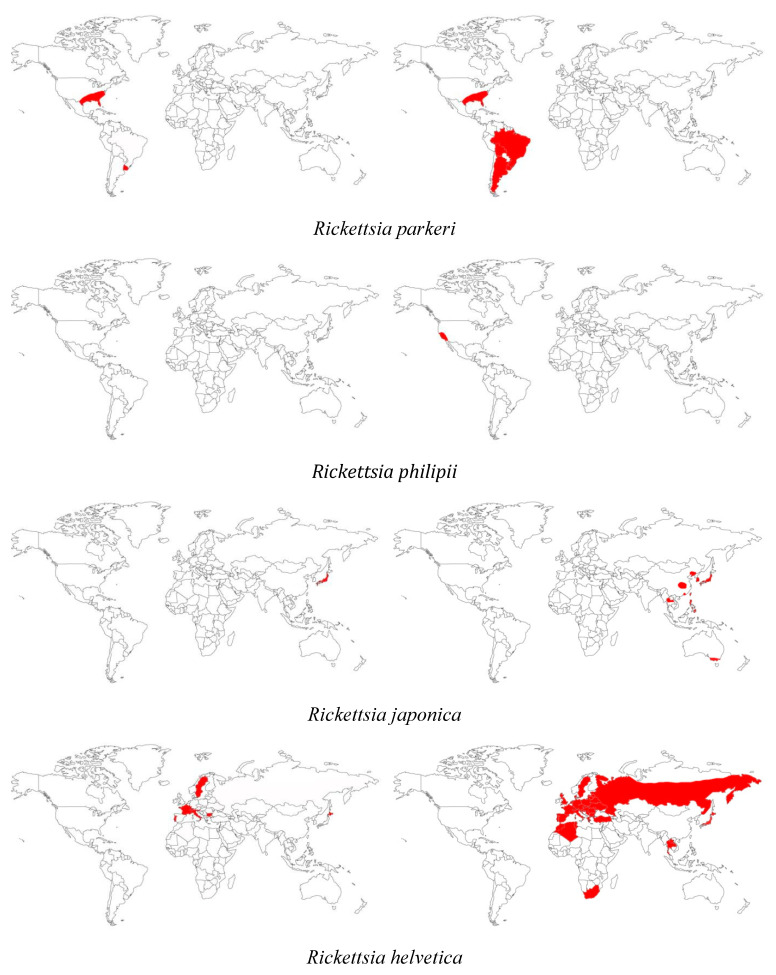

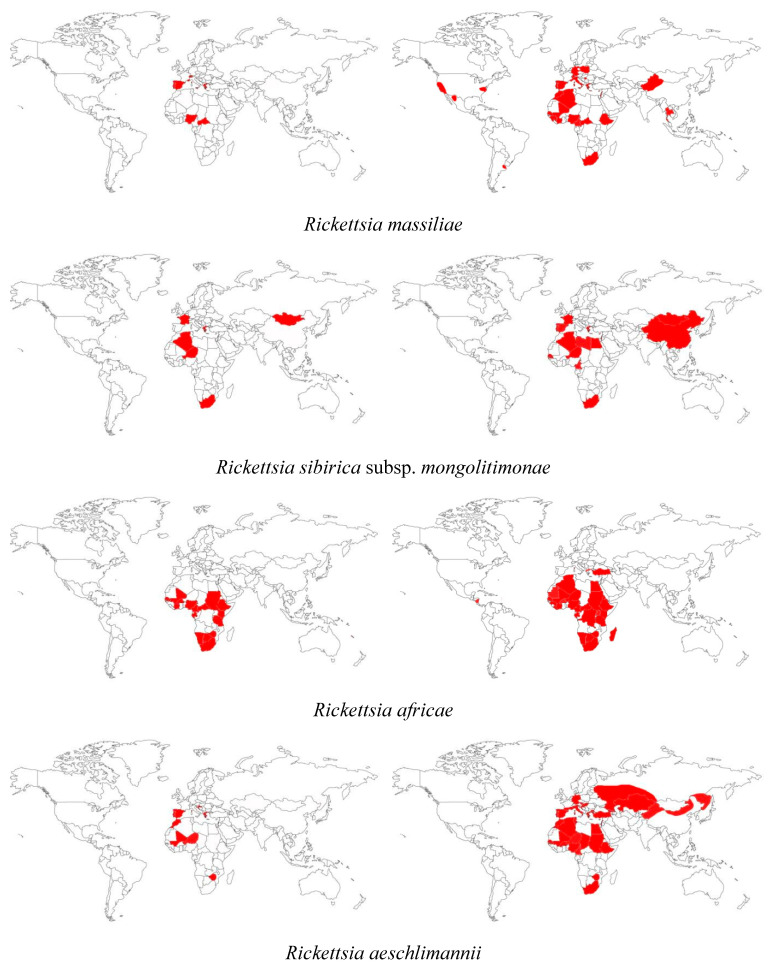

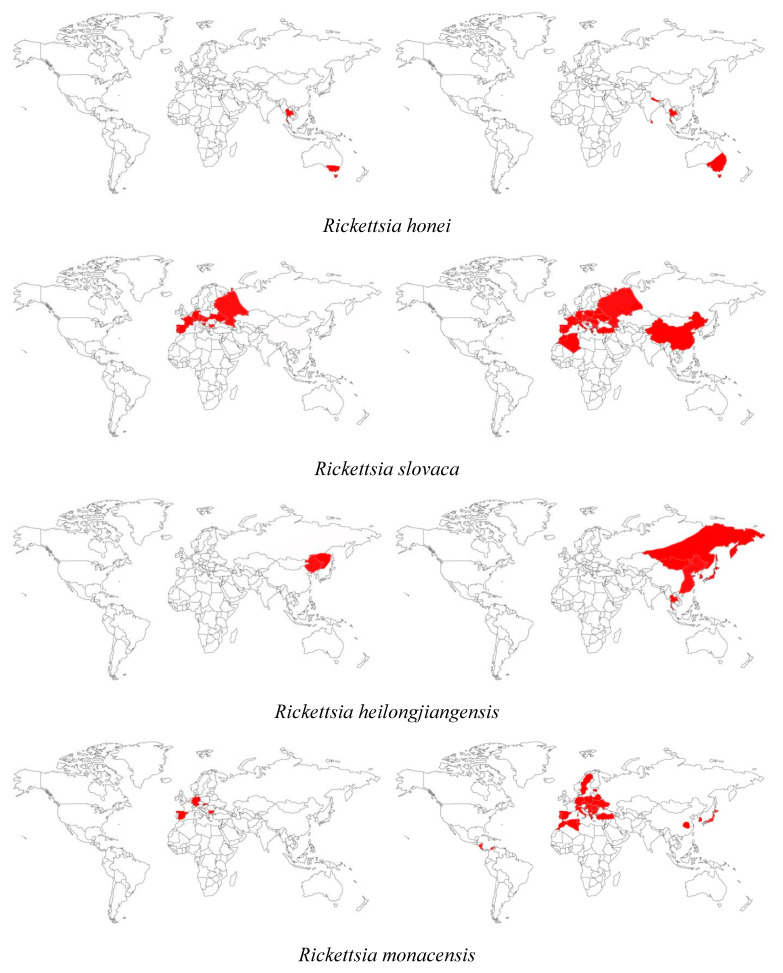

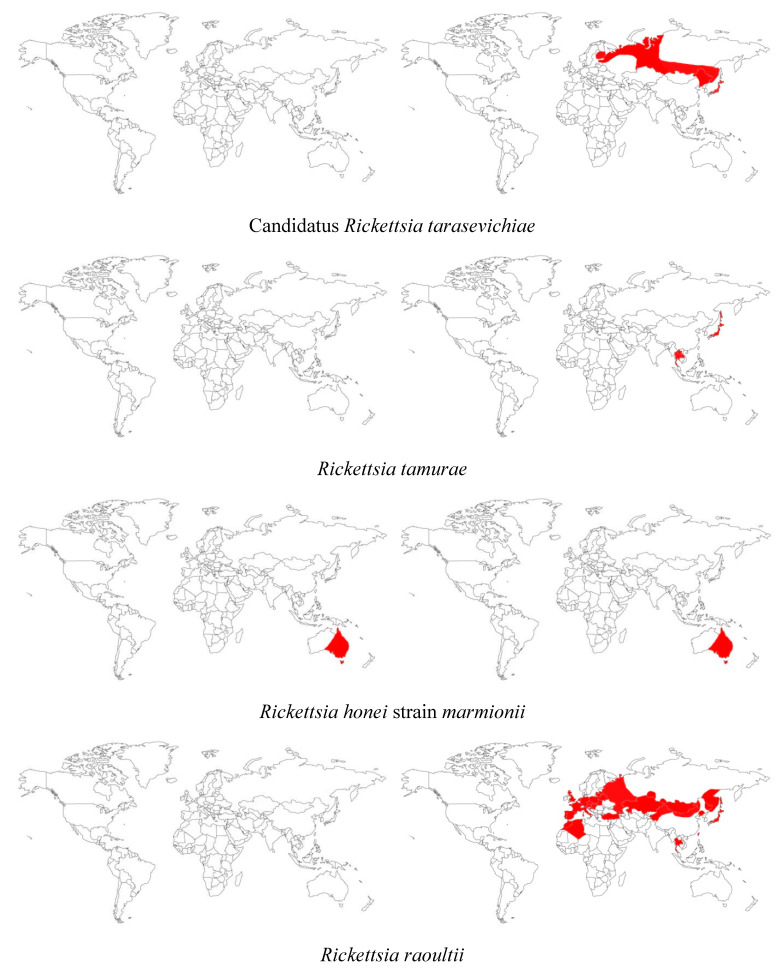

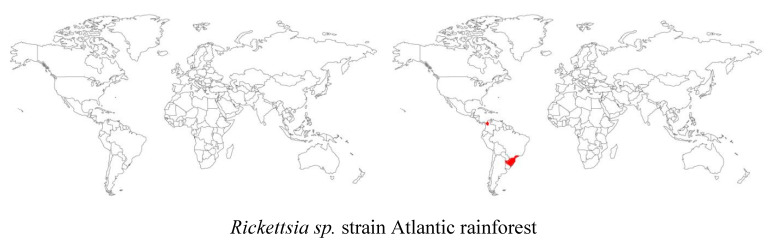
The occurrence of pathogenic tick-borne species belonging to the genus *Rickettsia* in the world at the beginning of the 21st century compared to now. On the left—the state of knowledge in 2005. On the right—the state of knowledge in 2019 [1,4,17,18,19,20,21,22,23,24,25,26,27,28,29,30,31,33,35,36,38,39,41,42,43,44,45,46,47,56,98,99,100,101,102,103,104,105,106,107,108,109,110,111,112,113,114,115,116,117,118,119,120,121,122].

New cases of rickettsioses and new locations where the presence of bacteria of the genus *Rickettsia* are recognized are still being identified. The variety and rapid evolution of the distribution and density of ticks and diseases which they transmit shows us the scale of the problem. It is presented below what the distribution of pathogenic species of the genus *Rickettsia* looks like in the world in comparison to the beginning of our century (Figure 2).

In comparison to the data from the beginning of the 21st century, the current state of knowledge increases the number of pathogenic *Rickettsia* species from 18 to 26. It should be noted that some of these new pathogens were already known in 2005, but they were not considered to be pathogenic (*R. monacensis, R. montanensis, R. conorii* subsp. *indica*), while others were discovered and described later (*R. philipii,* Candidatus *R. tarasevichiae, R. tamurae, R. raoultii, Rickettsia sp.* strain Atlantic rainforest).

## 3. Diagnostics

Clinical symptoms of rickettsioses from the spotted fever group (SFG) generally start from four to ten days after a tick bite and usually include fever, headache, myalgia, rash, enlarged lymph nodes and in most cases the occurrence of an eschar at the tick bite site. However, many symptoms vary depending on the type of rickettsiae, and typical symptoms may be absent or overlooked. Therefore, the diagnosis of rickettsioses is a big challenge for both physicians and diagnostic laboratories and requires cooperation [1].

Rickettsial diseases are very difficult to diagnose. There are several diagnostic methods used to identify rickettsioses, including serological methods, cell cultures, histochemical, immunohistochemical, and molecular methods [1]. The best material for diagnostic tests are those with high concentration of bacteria, so eschars, where swabbing is fast, comfortable and painless for a patient [123] and biopsy of skin with rash, which is an invasive and painful method, cannot be carried out in any doctor’s surgery. Blood, serum, or plasma samples may also be used. However, several important points should be taken into account when collecting these samples. First, unless it is an extreme infection, the level of bacteria in blood is lower than in tissue samples from rash or eschars, and therefore there is an increased risk of false negative results [124]. It should be noted that regardless of the sample, antibiotic treatment before sampling reduces the effectiveness of the diagnosis either through culture, serology, or molecular methods [125].

Serological tests are the easiest, most commonly used, and most widely available method of diagnostics of rickettsioses enabling the detection of antibodies against *Rickettsia* in serum or plasma samples. Nevertheless, antibodies are detected only seven to 15 days after the occurrence of the disease, and in the cases of *R. africae* it can even be 25 to 28 days [1]. Immunofluorescence assays (IFA) are reference tests, also considered as the “gold standard” for the detection of rickettsial diseases by measures of levels of IgG and IgM antibodies. Other serological methods include the Weil-Felix test, enzyme-linked immunosorbent assay (ELISA) and Western blot analysis [124]. The disadvantage of serology is that available tests are not specific enough to detect individual *Rickettsia* species. Serological tests are also not sensitive in the early stage of an acute illness when most patients look for medical help [71].

By using cell cultures, rickettsiae can be detected 48 to 72 h after inoculation by the infected tick. This technique is crucial for identifying new species enabling genetic descriptions, physiological analyzes, improvement of diagnostic tools and testing of susceptibility of bacteria to antibiotics. Although the number of laboratories adequately equipped for *Rickettsia* cultures is increasing, the isolation of the bacteria remains difficult and only few reference centers are able to do it. In addition, skin biopsy samples should be taken before treatment in the early stage of the disease, and cultures must be initiated immediately after sampling [1]. The material that can be used in this technique includes skin biopsy, heparinized blood, or hemolymph from a vector. Due to the difficult nature of cultures and the need to conduct it in a specialized laboratory, it is often recommended to apply cultures only in cases where the disease is so serious that the recognition of the species is decisive for the patient’s life [126].

The fastest method eliminating the delay in diagnostics are molecular tools that enable both a quick and convenient detection and identification of rickettsiae. Molecular methods are faster and can be used earlier than serology. Using the PCR technique, it is possible to detect the presence of bacteria in the blood, swabs, biopsies, and vectors [1]. In the diagnostics of rickettsiae, nested PCR and real-time PCR methods are used [124,127,128]. The most frequently used markers for the diagnostics of rickettsiae are fragments of *gltA*, *ompA,* and *ompB* genes [128].

**Figure 3:**
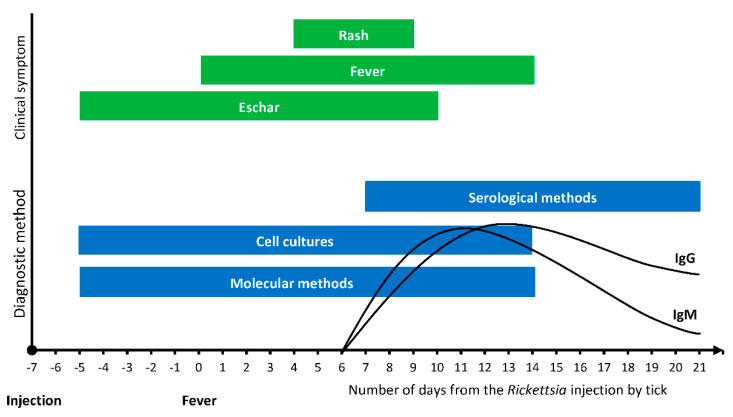
The usefulness of diagnostic methods in the course of tick-borne rickettsioses [124].

Each method of diagnosin rickettsial infections has its advantages and disadvantages. Cultures and molecular methods can be used for diagnosis in the early stage of acute infection. Serology can only be used as a diagnostic method when IgM and IgG become detectable, which occurs only about 10 days after inoculation. Antibodies become detectable at the same time, however, higher levels of IgM have been reported in the earlier stage, while IgG levels remain high for a longer time. Many researchers conclude that the combination of serological and molecular methods is the most effective way to diagnose the infection and to determine which *Rickettsia* species is present in a patient (Figure 3) [124].

Another helpful tool in identifying rickettsioses may be the recognition of which tick species attacked us. Ticks can therefore be used as an indirect diagnostic tool. Individual species of rickettsiae are transmitted by specific species of ticks (Table 1). Therefore, by recognizing a tick species, we are able to narrow down the potential pathogenic target. Thus, the identification of a tick species is clinically helpful because it is able to inform a doctor about illnesses that may have been transferred. Tick species can be morphologically identified using taxonomic keys that should be created for local geographic areas. However, the identification of tick species requires some entomological knowledge and may be difficult or even impossible in the case of immature or damaged ticks. If a tick is a recognized vector for a particular disease, this information can be a valuable suggestion during a medical interview.

Health workers practicing in regions where the incidence of tick-borne diseases, including rickettsial ones, is historically low, may have a problem distinguish these diseases from other clinically similar and more common infectious and non-infectious syndromes. Tick-borne rickettsial diseases are usually sporadic, and the identification of these infections requires a high rate of clinical suspicion, especially in environments where infections have not been previously recognized. Therefore, it is important to have knowledge about the epidemiology of rickettsioses transmitted by ticks, including their distribution and seasonality [7]. 

## 4. Treatment

If the symptoms typical for rickettsioses are recognized, treatment should be started immediately, before the laboratory confirmation of the diagnosis [129]. The decision regarding the treatment of rickettsial diseases should never be delayed while awaiting laboratory confirmation. Delay in treatment can lead to serious disease and long-term consequences or death [7].

The first-choice medicine for infections caused by rickettsiae is doxycycline. It is the obvious choice, especially in children with severe courses of rickettsiosis, because it shows a very good tolerance. An alternative to doxycycline in the treatment of certain rickettsioses is josamycin and new macrolide compounds such as clarithromycin and azithromycin. They can be used in pregnant women, but under close observation and when there is lack of severe symptoms. Telithromycin also seems to be highly active in vitro, but there is lack of in vivo data; therefore, these medicines should be further evaluated [129]. In any case where there is suspicion of rickettsiosis transmitted by a tick, early empiric antibiotic therapy should be prescribed [1].

The recommended dose of doxycycline for the treatment of rickettsiosis is 100 mg twice daily (either orally or intravenously) for adults and 2.2 mg per kilogram of body weight twice daily (orally or intravenously) for children weighing less than 45 kg. Oral therapy is suitable for patients with early-stage disease who can be treated in the ambulatory way. Intravenous therapy may be indicated for seriously ill patients requiring hospitalization. The duration of treatment is at least three days after the disappearance of fever and until clinical improvement. Usually, the minimum total treatment time is five to seven days. A severe or complicated disease may require a longer treatment period. The American Pediatric Academy and the CDC recommend the use of doxycycline as the choice of treatment in children of all ages with suspected rickettsial disease [7].

## 5. Supervision

One of the ways to understand the dynamics of tick-borne diseases is the systematic recording of the infection cases. This can be achieved by implementing a special surveillance system based on declarations from general practitioners and/or infectious disease specialists. In many countries, there is an obligation to register tick-borne diseases and the results are available in the form of systematic national reports. In addition, ECDC (European Centre for Disease Prevention and Control) collects information on rickettsioses from European countries, and CDC (Centers for Disease Control and Prevention) from the United States. However, the collected data is incomplete and unreliable, and it is affected by the fact that most definitions of tick-borne diseases are not standardized. This is partly due to the heterogeneity of epidemiological conditions, but also due to different availability of diagnostic tests in individual countries and various assessments of their importance by national health authorities. Such an attitude is also not very effective due to the fact that in areas of low prevalence of rickettsial diseases, milder forms are so rare that they may not be identified by physicians at all. Without detailed surveillance data, the increase in the number of new cases may only indicate an increase in the number of doctors interested in this subject matter or using improved diagnostic tools, rather than an actual increase of the risk of illness [130]. Another way to assess the risk of tick-borne diseases in humans is to monitor the circulation of pathogens in wild animals, domestic animals or farm animals. Serological tests of animal blood and data obtained on the basis of collected ticks from these hosts are both very important. That data, apart from the number and distribution of ticks, can give valuable information on the presence of pathogens in foraging ticks, and consequently, an assessment for rickettsiosis infection after a tick bite. Field studies conducted by entomologists are another very valuable database. While they are collecting material for research, they can analyze the distribution of ticks in relation to biotopes, landscapes, climates, altitude, and urbanization. Such collections provide descriptions of the spread of basic tick-borne vectors and help to characterize their biotopes, and at the same time they potentially identify areas and periods of exposure to tick bites. In September 2009, thanks to the ECDC support, the Vbornet project was created, which mapped the distribution of ticks *I. ricinus*, *I. persulcatus, H. marginatum,* and *D. reticulatus* based on published historical data, and the works have been confirmed by experts from countries that have participated in this process. However, this project existed actively only until 2012, and in June 2014 it was replaced by a project called VectorNet, which is a joint initiative of the European Food Safety Authority (EFSA) and European Centre for Disease Prevention and Control (ECDC). VectorNet’s activity continues to this day and supports the collection of data on vectors and pathogens in vectors related to both human and animal health. The aim of ECDC and EFSA is to maintain a common database on the presence and distribution of vectors and pathogens in vectors in Europe and the Mediterranean Basin through the development of a network of experts and organizations in the field of medicine and veterinary. Other regions of the world are also creating their monitoring program, and such an example is the TickNET program created in the United States in 2007. This program integrates and supports cooperation between the State Health Departments, Academic Centers and Centers for Disease Control and Prevention in the field of surveillance, research, education, and prevention of diseases transmitted by ticks. However, the number of ticks may change from year to year depending on climatic conditions, therefore research must be carried out at different times in subsequent years. In addition, microclimate conditions are important and different biotopes should be studied in the same area. Information on the identification of pathogens should provide information on the circulation of the pathogen in a given zone and allow for the detection of new pathogens in these areas. Although entomological studies provide important information on potential vectors, their density, and infectivity, these field methods are labor-intensive and expensive, and the results are limited only to the monitored areas.

A difficult but noteworthy approach is the possibility of creating mathematical models, which thanks to data in the form of climatic changes, changes in biotopes, in human activity and animal numbers could identify tick populations and at the same time regions and risk periods for tick-borne diseases [130]. Although the prognostic models have to take into account many parameters, the first trials have already been carried out, and the data is promising [131,132,133]. 

Another interesting approach proposed by scientists is the estimation of exposure to ticks through serological tests detecting antigens on proteins found in the tick’s saliva. Tests were carried out for the first in the 90s of the last century. These studies emphasize the potential of the host’s immune response against the proteins of the vector’s saliva, which can be used as biomarkers for tick exposure. In addition, there was also a significant reduction in the response during the absence of exposure to ticks for several months (from October to January). This finding reveals that the host’s immune response is temporary and therefore it is useful for investigating seasonal exposure according to the density of ticks [134,135,136]. What is more, some tick saliva proteins can be unique at the level of species or genus, which gives a more precise identification tool [137]. To sum up, a tool that detects antigens for proteins found in the tick’s saliva is a promising method that can improve other previously developed tools to assess the risk of tick bite exposure. This newly developed method can be used in both epidemiological and entomological studies to characterize endemic areas, seasonal variations and potential areas of exposure to tick-borne diseases [130].

It is difficult to control tick-borne diseases due to complex epidemiology, including various species of ticks and different hosts. Doctors and vets can also follow various pathways. Currently, it is clear that an integrated approach is required to control tick-borne diseases. It is necessary to unite the medical and veterinary branches in order to better manage this important group of diseases, filling gaps in communication between doctors and vets in order to accelerate diagnosis, accelerate decisions about treatment and implement preventive measures. The basic principle is to increase communication and information exchange between doctors and vets in all aspects of tick-borne diseases at the local, regional, and national level. Wild and domestic animals as well as ticks associated with them may be involved in the epidemiology of tick-borne diseases. Even if domestic animals are not the main reservoirs of rickettsioses, they can bring ticks infected with these pathogens to human flats, and both vets and physicians should keep this in mind [138].

Surveillance systems are necessary to study the changing epidemiology of tick-borne rickettsial diseases and to develop effective prevention strategies and public health activities. As the new data on the occurrence of ticks and zoonotic diseases in urban areas are being collected, there is an urgent need to create an effective and environmentally friendly system of protection for urban residents against tick attacks and associated infections. In order to carry out this task successfully, it is necessary to systematize our knowledge about various aspects of ticks in cities and towns and to understand the factors affecting their presence.

## 6. Prevention

Prevention of tick attacks is generally based on the use of chemicals, mainly acaricides. Several active ingredients with a killing and/or repellent effect are commercially available, and the most commonly used repellent against ticks is DEET (*N*,*N*-diethyl-3-methylbenzamide) at a concentration of 10% to 35%, EBAAP (ethyl-butylacetylaminopropionate), picaridin, and permethrin, which kills tick and should only be used for clothing. Active ingredients may be available in different forms. In the protection of people, mainly sprays are used, in the protection of pets, impregnated collars, shampoos and sprays are used, and in the protection of livestock, pour-on solutions are used [139,140].

The best way of protection against most rickettsial diseases is prevention from vector bites. Wearing long trousers slipped into shoes, long socks and full shoes, and the use of tick repellents are effective tools to reduce the risk of tick bites and transmission of the pathogen. Avoiding tick bites and removing ticks immediately remains the best strategy for disease prevention. People visiting areas where ticks may occur should always check themselves, their children and their pets, remove any ticks immediately and reduce the risk of transmission of the pathogen. Baths or showers as well as detailed physical control shortly after exposure to a tick habitat are recommended to eliminate unattached ticks and remove the attached ones. Scalp, underarms and groins, as well as the area of socks and along the waistline are the sites where ticks most often penetrate the human skin. Pets should also be checked in detail because they can bring ticks home, which increases the risk of human exposure. Ticks on dogs are commonly found around the ears, between the toes and in the armpits and groins [7].

Ticks attached to the skin should be removed immediately. The preferred method of removal is to grasp the tick close to the skin’s surface with fine-tipped tweezers or other commercially available devices and pull it upward with steady pressure. Care should be taken to avoid crushing the body of the tick to prevent contact of potentially infectious tick fluids with the skin. Petrol, kerosene, vaseline, nail polish remover, or lighted matches should never be used to remove ticks. A wide range of devices has been introduced on market to help remove the attached ticks, but their effectiveness has not been proven to be better than normal tweezers. If it is possible, removing the ticks with fingers should be avoided, because we can be exposed to the bloodstream fluids from the body of the tick, which may contain pathogens. Moreover, the removed ticks should not be crushed with fingers. After removing the tick, the bite site should be thoroughly cleaned with soap and water, alcohol, iodine peeling, or with the use of antiseptics. Hands of people who could have touched the tick should also be carefully washed, especially before touching face or eyes [7].

Bacteria of the genus *Rickettsia* are usually observed in the salivary glands of ticks and can be transmitted even before starting the food intake. Therefore, the attached ticks should be removed as soon as possible because the risk of infection is minor only in the first six hours after the bite [6].

Walking on cleared trails, avoiding vegetation and creating zones safe from ticks in courtyards and gardens can help reduce the risk of tick bites [7].

## 7. Conclusions

The growing interest and awareness of clinicians, combined with better and better diagnostic methods, allows for the discovery of new organisms belonging to the genus *Rickettsia*, including those pathogenic ones. Because of ecotourism, which becomes more and more popular, as well as travelling around the world, the incidence of tick-borne rickettsioses will most likely increase in the near future. According to the data of the World Tourism Organization (UNWTO) the number of international tourists in 2017 reached 1323 billion people, which is a 7% (84 million) increase in comparison to 2016. The average increase in international tourism for the last eight years was 4% per year. Since 2008, the number of travelers has increased by 393 million. In the coming years, a systematic increase in the number of travelers is predicted. The risk of tick-borne diseases is increasing worldwide, and this situation seems to be driven by several factors. Populations of wild animals can naturally migrate, transferring ticks and pathogens that live in them from one area to another. Travelers can also play a role in transferring an exotic tick or disease to areas previously free from them. Tick-borne diseases are increasingly diagnosed in travelers returning from endemic areas, which may be a diagnostic challenge for less experienced physicians in non-endemic areas.

The fact is that areas with tick-borne disease incidence, including rickettsioses, are increasing, as well as the number of new cases. Joining the efforts of health professionals is crucial to remedy this situation and reduce the current burden of infection and death cases. Change in attitude and better communication between physicians and vets may play a key role in this process, and it is clear that there is a need for a common and uniform approach to manage tick-borne diseases in a better way. The recognition of new cases is a challenge, and the exchange of information between doctors and vets is beneficial not only for them, but above all for patients. In this changing scenario of tick-borne diseases, continuous education is essential. Physicians and vets must be aware of new discoveries and developments that will improve their clinical practice. All over the world, national and local solutions dedicated to this issue should be promoted, in which vets, physicians and scientists sit at one table. Only a multidisciplinary approach to the problem can be successful in protecting society from tick-borne diseases.

Scientists should try to create new information databases on current and emerging cases of tick-borne diseases. Education is the key to control the risk and public health. Decision makers, scientists, physicians, vets, dog owners, farmers, travelers, etc. should be aware of the danger of ticks and how to fight with them.

## Figures and Tables

**Table 1:** List of currently known species of ticks transmitting pathogenic bacterial species of the genus *Rickettsia* [1,4,16,17,18,19,20,21,22,23,24,25,26,27,28,29,30,31,32,33,34,35,36,37,38,39,40,41,42,43,44,45,46,47,48].

Pathogenic Species *Rickettsia*	Tick Species Identified as a Vector
*Rickettsia aeschlimannii*	*Amblyomma tigrinum, A. variegatum,* *Dermacentor marginatus, D. nuttalli,* *Haemaphysalis concinna, H. japonica, H. punctata,* *Hyalomma aegyptium, H. anatolicum excavatum, H. detritum detritum, H. detritum, H. dromedari, H. excavatum, H. impeltatum, H. lusitanicum, H. marginatum marginatum, H. marginatum rufipes, H. marginatum, H. truncatum,* *Ixodes ricinus,* *Rhipicephalus annulatus, R. appendiculatus, R. bursa, R. evertsi evertsi, R. sanguineus, R. turanicus,*
*Rickettsia africae*	*Amblyomma cohaerens, A. compressum, A. eburneum, A. gemma, A. hebraeum, A. lepidum, A. loculosum, A. ovale, A. variegatum,* *Hyalomma aegyptium, H. dromedarii, H. impeltatum, H. marginatum rufipes,* *Rhipicephalus annulatus, R. decoloratus, R. evertsi, R. geigyi, R. sanguineus,*
*Rickettsia australis*	*Ixodes cornuatus, I. holocyclus, I. tasmani,*
*Rickettsia conorii*	*Haemaphysalis leachi, H. punctaleachi,* *Rhipicephalus bursa, R. evertsi evertsi, R. mushamae, R. pumilio, R. sanguineus, R. simus, R. turanicus,*
*Rickettsia conorii* subsp. *indica*	*Rhipicephalus sanguineus*, *R. turanicus,*
*Rickettsia conorii* subsp. *israelensis*	*Rhipicephalus sanguineus,*
*Rickettsia conorii* subsp. *caspia*	*Rhipicephalus pumilio, R. sanguineus,*
*Rickettsia heilongjiangensis*	*Dermacentor niveus, D. nuttalli, D. silvarum,* *Haemaphysalis concinna, H. flava, H. japonica douglasi, H. japonica, H. longicornis, H.verticalis, Hyalomma asiaticum kozlovi,* *Ixodes persulcatus,* *Rhipicephalus pumilio,*
*Rickettsia helvetica*	*Dermacentor reticulatus,* *Ixodes arboricola, I. festai, I. hexagonus, I. persulcatus, I. ricinus, I. trianguliceps,*
*Rickettsia honei*	*Bothriocroton hydrosauri,* *Haemaphysalis novaeguineae,* *Ixodes sp.,*
*Rickettsia honei* strain *marmionii*	*Haemaphysalis novaeguineae,*
*Rickettsia japonica*	*Dermacentor taiwanensis,* *Haemaphysalis cornigera, H. flava, H. formosensis, H. hystricis, H. longicornis,* *Ixodes ovatus,*
*Rickettsia massiliae*	*Amblyomma sylvaticum,* *Dermacentor variabilis,* *Haemaphysalis paraleachi,* *Ixodes ricinus,* *Rhipicephalus bursa, R. evertsi, R. guilhoni, R. lunulatus, R. muhsamae, R. praetextatus, R. pusillus, R. sanguineus, R. senegalensis, R. sulcatus, R. turanicus,*
*Rickettsia monacensis*	*Amblyomma dissimile,* *Dermacentor variabilis,* *Ixodes boliviensis, I. persulcatus, I. ricinus, I. sinensis,* *Rhipicephalus sanguineus*
*Rickettsia montanensis*	*Amblyomma americanum,* *Dermacentor andersoni, D. variabilis,*
*Rickettsia parkeri*	*Amblyomma americanum, A. maculatum, A. tigrinum, A. triste, A. debitatum,* *Dermacentor variabilis,*
*Rickettsia philipii*	*Dermacentor occidentalis,*
*Rickettsia raoultii*	*Amblyomma testudinarium,* *Dermacentor everstanii, D. marginatus, D. niveus, D. nuttalli, D. reticulatus, D. silvarum,* *Haemaphysalis concinna, H. japonica, H. lagrangei, H. longicornis, H. ornithophila, H. shimoga, H. verticalis,* *Hyalomma asiaticum, H. marginatum,* *Ixodes persulcatus, I. ricinus,* *Rhipicephalus sanguinaus*
*Rickettsia rickettsii*	*Amblyomma americanum, A. aureolatum, A. cajennense, A. imitator,* *Dermacentor andersoni, D. nitens, D. occidentalis, D. variabilis,* *Haemaphysalis leporispalustris,* *Rhipicephalus sanguineus,*
*Rickettsia sibirica* subsp. *mongolitimonae*	*Dermacentor marginatus, D. nuttalli, D. reticulatus, D. silvarum, D. sinicus,* *Haemaphysalis concinna, H. erinacei, H. yeni,* *Hyalomma aegyptium, H. anatolicum excavatum, H. asiaticum, H. truncatum,* *Ixodes persulcatus,* *Rhipicephalus pusillus, R. turanicus*
*Rickettsia sibirica* subsp. *sibirica*	*Dermacentor nuttalli, D. silvarum, D. sinicus* *Ixodes kaiseri,*
*Rickettsia slovaca*	*Dermacentor marginatus, D. nuttalli, D. reticulatus,* *Hyalomma aegyptium,* *Rhipicephalus sanguineus,*
*Rickettsia tamurae*	*Amblyomma testudinarium,*
*Rickettsia sp.* strain Atlantic rainforest (lub strain Bahia)	*Amblyomma aureolatum, A. dubitatum, A. ovale,* *Rhipicephalus sanguineus,*
Candidatus *Rickettsia tarasevichiae*	*Dermacentor reticulates,* *Haemaphysalis concinna* *Ixodes persulcatus, I. ricinus,*
